# The circadian clock transcriptional complex: metabolic feedback intersects with epigenetic control

**DOI:** 10.1111/j.1749-6632.2012.06649.x

**Published:** 2012-07-26

**Authors:** Selma Masri, Loredana Zocchi, Sayako Katada, Eugenio Mora, Paolo Sassone-Corsi

**Affiliations:** Center for Metabolism and Epigenetics, U904 Inserm “Epigenetics and Neuronal Plasticity,” School of Medicine, University of CaliforniaIrvine, California

**Keywords:** circadian clock, epigenetics, metabolism

## Abstract

Chromatin remodeling is a prerequisite for most nuclear functions, including transcription, silencing, and DNA replication. Accumulating evidence shows that many physiological processes require highly sophisticated events of chromatin remodeling. Recent findings have linked cellular metabolism, epigenetic state, and the circadian clock. The control of a large variety of neuronal, behavioral, and physiological responses follows diurnal rhythms. This is possible through a transcriptional regulatory network that governs a significant portion of the genome. The harmonic oscillation of gene expression is paralleled by critical events of chromatin remodeling that appear to provide specificity and plasticity in circadian regulation. Accumulating evidence shows that the circadian epigenome appears to share intimate links with cellular metabolic processes. These notions indicate that the circadian epigenome might integrate tissue specificity within biological pacemakers, bridging systems physiology to metabolic control. This review highlights several advances related to the circadian epigenome, the contribution of NAD^+^ as a critical signaling metabolite, and its effects on epigenetic state, followed by more recent reports on circadian metabolomics analyses.

## Circadian rhythms: systems biology

A wide variety of physiological functions, including sleep–wake cycles, body temperature, hormone secretion, locomotor activity, and feeding behavior depend on the circadian clock—a highly conserved system that enables organisms to adapt to common daily changes, such as the day–night cycle and food availability.^1^ Based on evidence accumulated during several decades, it is safe to conclude that circadian rhythms represent one of the most clear examples of systems biology.^2^ Our understanding of circadian rhythms indicates that these cyclic events are self-sustained and centrally controlled, suggesting a complex and intricate biological timing mechanism that governs our daily behavior. Disruption of circadian rhythms has been linked to numerous diseases, including sleep disorders, depression, metabolic syndrome, and more recently an emerging role in tumorigenesis.^3^,^4^

In mammals, the anatomical structure in the brain that governs circadian rhythms is a small area consisting of ∼15,000 neurons localized in the anterior hypothalamus, called the suprachiasmatic nucleus (SCN).^5^,^6^ This “central pacemaker” in the SCN receives signals from the environment and coordinates the oscillating activity of peripheral clocks that are located in almost all tissues.^1^,^7^–^9^ One important feature of the circadian clocks is that they are self-sustained: circadian oscillations intrinsic to each cell can occur autonomously, without any environmental signals. However, because the period of oscillation is not exactly 24 h, the endogenous clock needs to be synchronized by external cues, a process called *entrainment*. External cues (also known as *zeitgebers*) reset the system daily and thereby prevent the endogenous clock from free-running out of phase. The predominant external cue of the central clock is light.^10^ In mammals, specialized cells in the retina detect the light signal that is then transmitted to the SCN via the retinohypothalamic tract (RHT).^11^–^13^ At the level of SCN neurons, the light signal stimulates a cascade of signaling pathways that lead to the activation of a transcriptional program that involves immediate early genes and clock-controlled genes (CCGs). These gene expression events are associated with specific histone modifications leading to chromatin remodeling.^14^ Peripheral tissues also contain functional circadian oscillators that are self-sustained at the single-cell level, but they do not respond to light–dark cycles and appear to require other physiological stimuli in order to sustain their circadian rhythms.

The systemic control of the central SCN clock over peripheral clocks necessitates a hierarchical network to maintain proper biological timing events, and several studies have elegantly demonstrated this idea. Lesions of the rodent SCN disrupt the circadian periodicity in peripheral tissues, whereas SCN transplantation into SCN-ablated arrhythmic animals restores this disfunction.^5^,^15^ Additional experiments in which the transplantation approach was applied to peripheral tissues demonstrated a hierarchical dominance of the SCN over clocks in peripheral tissues.^16^ To date, however, the means by which the SCN communicates with peripheral tissues to sustain and synchronize their cycles is still not clear. Several observations support the idea that communication may be exerted by a combination of neuronal signals through the autonomic nervous system and humoral factors, of which glucocorticoids, and retinoic acid are the most likely candidates.^3^,^17^ In addition, expression of the SCN-secreted protein prokineticin 2 (PK2) is light sensitive, and levels of this protein are likely to regulate behavior and locomotor activity in mice, presumably through PK2 receptors (PKR2) found in surrounding regions of the brain.^18^ Similarly, transforming growth factor alpha (TGF-α) is another output signal of the SCN that has been implicated in sleep and locomotor activity by binding epidermal growth factor receptors found in the hypothalamic subparaventricular zone.^19^ Furthermore, peripheral rhythms in mammals are affected by other SCN-independent stimuli.^9^ Although light is the main stimulus that entrains the central pacemaker, peripheral clocks can themselves be entrained by food,^20^ probably through modifications of hormonal secretion or metabolite availability. Restricted access to food can reset the phase of peripheral oscillators, with little if any effects on the SCN central pacemaker.^21^ These notions underscore the intimate links between the circadian clock and cellular metabolism.^3^,^22^

Another important environmental cue is temperature.^23^ Temperature compensation is one of the most prominent features of the circadian system as it allows the integration of moderate variations in ambient temperature that do not affect the period length of circadian oscillation. Nevertheless, low-amplitude temperature cycles can synchronize the circadian clocks in peripheral tissues in mammals, independently of the central clock.^24^

## The circadian transcriptome

At the heart of the molecular network that constitutes the circadian clock are the core transcription factors CLOCK and BMAL1 that heterodimerize and direct transcriptional activation of CCGs, by binding to E-box sites within their promoters. Among these CCGs, CLOCK and BMAL1 also direct transcription of their own repressors, period (PER), and cryptochrome (CRY) family members, creating a tightly self-regulated system.^4^ During the day, transcription of PER and CRY is high, leading to protein translation of the circadian repressors, and resulting in formation of the inhibitory complex with CLOCK and BMAL1 that abolishes transcription of CCGs. The degradation of PER and CRY alleviates transcriptional repression and allows CLOCK:BMAL1-mediated transcription to again proceed, establishing an oscillatory rhythm in circadian gene expression. An additional level of circadian regulation exists with the orphan nuclear receptors RORα and REV-ERBα that activate and repress transcription of the *Bmal1* gene, respectively.^25^,^26^ Furthermore, the possibility that the clock protein may be regulated in a posttranslational manner, as in the case of SUMOylation of BMAL1,^27^ adds an additional level of regulation of the clock machinery.

While the basic molecular organization and conceptual design of these autoregulatory loops are common to both SCN and peripheral tissues, it is intuitive that circadian function and output of SCN, liver, or skeletal muscle are vastly divergent, begging the question on how the pacemakers intrinsic to these tissues may differ. Indeed, the property of circadian synchronicity in culture is unique to SCN neurons: cultured cells from peripheral tissues, although each has a sustained circadian cycle, do not display concerted oscillations.^28^ On the other hand, it is reasonable to speculate that tissue-specific transcriptional regulators may contribute or intersect with the clock machinery. Several genome-wide array analyses have been centered on determining the proportion and specificity of cycling transcripts.^29^ The first remarkable finding indicated that ∼10% of all expressed genes in any tissue are under circadian regulation.^1^,^28^,^30^,^31^ This unexpectedly high proportion of circadian transcripts suggests that the clock machinery may direct widespread events of cyclic chromatin remodeling and consequent transcriptional activation/repression. Furthermore, genome-wide studies comparing the central SCN pacemaker and peripheral tissues, such as the liver, revealed that between 5% and 10% of cycling genes were identical in both tissue types.^32^,^33^ A recent analysis covering 14 mouse tissues identified ∼10,000 known genes showing circadian oscillations in at least one tissue. The number of common genes showing circadian oscillation in multiple tissues decreased drastically as the number of tissues included in the comparative analysis increased, with only 41 genes displaying circadian oscillation in at least 8 out of 14 tissues.^34^ These findings underscore the presence of molecular interplay between the core clockwork, which can be assumed to be common to all tissues, and cell-specific transcriptional systems. Taking into consideration the recent view of the mammalian circadian clock as a transcriptional network,^2^,^35^ through which the oscillator acquires plasticity and robustness, it is reasonable to speculate that the clock network contributes to physiological responses by intersecting with cell-specific transcriptional pathways. This notion has been demonstrated in the way the circadian machinery interplays with other signaling-responsive transcription factors, such as CREB.^36^

## Chromatin remodeling and epigenetic control of circadian expression

How does the complex organization of chromatin cope with the cyclic regulation of circadian genes? Several histone modifications contribute to chromatin remodeling and thereby to the control of a large array of nuclear processes.^37^,^38^ A number of histone modifications have been associated with distinct chromatin-based outputs. For example, position-specific modifications of the histone H3 N-terminal tail have been coupled to transcriptional regulation (Lys4 and Lys9/Lys14 acetylation, Ser10 phosphorylation), transcriptional silencing (Lys9 methylation), histone deposition (Lys9 acetylation), and chromosome condensation/segregation (Ser10/Ser28 phosphorylation). It is believed that specific signaling pathways lead to distinct histone modifications,^39^ suggesting that various physiological stimuli translate into differential chromatin remodeling events.^40^

**Figure 1:**
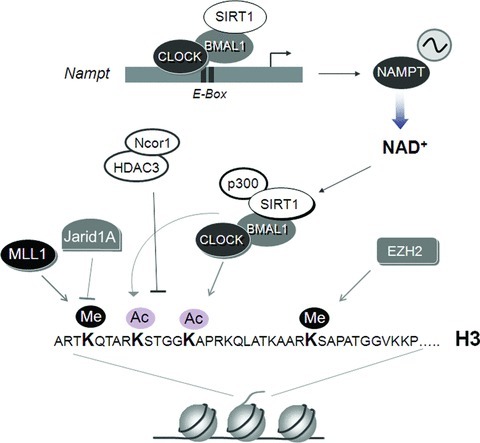
Linking cellular metabolism with the circadian clock transcriptional complex. A series of studies demonstrated that the circadian clock machinery controls the cyclic synthesis of NAD^+^ through control of the NAD^+^ salvage pathway.^55^,^56^ The gene encoding the enzyme NAMPT, the rate-limiting step in the NAD^+^ salvage pathway, contains E-boxes and is controlled by CLOCK-BMAL1. A crucial step in the NAD^+^ salvage pathway is controlled by SIRT1, which also contributes to the regulation of the *Nampt* promoter by associating with CLOCK-BMAL1 in the CLOCK chromatin complex.^29^ Oscillating levels of NAD^+^ also regulate chromatin remodeling events through SIRT1 and ultimately connect clock-dependent transcriptional control with a cellular metabolic pathway. MLL1 also directs the cyclic histone H3 Lys4 trimethylation event that is responsible for CLOCK:BMAL1 recruitment and permits circadian gene expression.^62^ Also included is the HDAC3 complex, which targets H3K9 acetylation in a circadian manner,^53^ EZH2,^63^ and Jarid1a.^50^ NAD^+^, nicotinamide adenine dinucleotide; ∼ indicates oscillation; Me, methylation; Ac, acetylation.

Histone acetylation has been shown to play a pivotal role in the modulation of chromatin structure associated with transcriptional activation.^41^–^45^ In support of this notion, a wide variety of nuclear proteins involved in transcriptional control possess intrinsic histone acetyltransferase (HAT) activity. We have found that one of these proteins is the master regulator CLOCK, whose HAT function is essential for circadian control.^46^ We have shown that chromatin remodeling is coupled to circadian clock function^14^ and that the protein CLOCK functions as an enzyme, which induces chromatin remodeling.^46^ This previously unforeseen activity of a core clock factor has several, far-reaching biological implications. CLOCK is a HAT, which preferentially modifies histone H3 in position Lys14, a site where addition of an acetyl group results in stimulation of gene expression (Fig. 1). Thereby, CLOCK acts as an enzyme that globally modifies genome functions, by inducing the opening of chromatin structure and allowing transcriptional activation. In addition, the enzymatic activity of CLOCK is not restricted to histones.^47^ Our findings indicate that CLOCK acetylates its own transcriptional partner, BMAL1. This modification occurs at one unique lysine residue in position 537 of the protein and is essential for circadian rhythmicity.^47^,^48^ Recent data indicate that the histone methyltransferase MLL1 directs the cyclic trimethylation of histone H3 Lys4 on circadian promoters, which subsequently mediates the recruitment of the CLOCK:BMAL1 complex to chromatin.^49^ Moreover, the demethylase Jarid1a is recruited with CLOCK/BMAL1 to circadian gene promoters and is involved in modulating the acetylation of histone H3 by inhibiting HDAC1, and subsequently enhancing transcription of the clock complex.^50^

The recent discovery that the activity of SIRT1, a longevity-associated protein belonging to a family of nicotinamide adenine dinucleotide (NAD^+^) activated histone deacetylases,^51^ oscillates in a circadian fashion broadens our knowledge about the communication between the circadian clock and metabolism. Yet this finding also reveals a void in our understanding about the interplay between the metabolic state of the cell and circadian control on the molecular level. SIRT1 counterbalances the HAT function of CLOCK by deacetylating both H3 Lys9/14 and BMAL1,^48^ as well as the deacetylation of the circadian regulatory protein PER2.^52^ SIRT1 demonstrates an oscillation in activity, impinging back on the circadian clock by altering BMAL1 acetylation and CLOCK:BMAL1-induced gene transcription.^48^,^52^ In addition to the deacetylase activity of SIRT1, HDAC3 has been reported to modulate chromatin marks in a circadian manner. HDAC3 is recruited to the genome in a rhythmic manner in mouse liver, and histone acetylation at H3K9 is inversely correlated with HDAC3 recruitment.^53^ Moreover, it was found by genome-wide ChIP-seq analysis that HDAC3 and Rev-erbα were co-localized at a number of common genes involved in lipid metabolism, amino acid metabolism, and carbohydrate metabolism in the mouse liver.^53^ A number of different lines of evidence suggest that an intricate relationship exists between chromatin state and cellular metabolism that is under the control of the circadian clock. What is quite intriguing is the bidirectional regulation between metabolism and epigenetics, suggesting that the circadian clock may control a complex network of feedback signals that we are only beginning to understand.

## NAD^+^ as a central circadian regulator

The discovery of metabolite oscillations during the yeast metabolic cycle,^54^ combined with evidence of circadian-directed sirtuin activity, allows speculation as to whether metabolites such as NAD^+^ themselves serve a preponderant role in the cellular link between metabolism and the circadian clock. Indeed, NAD^+^ itself is a critical signaling metabolite that is under the control of the circadian clock. Using accurate mass spectrometry/liquid chromatography measurements, our laboratory and others have confirmed this notion by demonstrating that NAD^+^ levels oscillate in serum-entrained MEFs and in liver.^55^,^56^ The circadian clock controls the expression of nicotinamide phosphoribosyltransferase (NAMPT), a key rate-limiting enzyme in the salvage pathway of NAD^+^ biosynthesis. CLOCK, BMAL1, and SIRT1 are recruited to the *Nampt* promoter in a time-dependent manner. The oscillatory expression of NAMPT is abolished in *clock/clock* mice, which results in drastically reduced levels of NAD^+^ in MEFs derived from these mice.^55^ These results make a compelling case for the existence of an interlocking classical transcriptional feedback loop that controls the circadian clock, with an enzymatic loop in which SIRT1 regulates the levels of its own cofactor.

The oscillation of NAD^+^ levels begs the question of whether the activity of other NAD^+^-dependent enzymes may be regulated in a circadian manner. In this respect, one class of enzymes appears to occupy a privileged position: the poly(ADP-ribose) polymerases (PARPs), which have been shown to functionally interact with SIRT1.^57^ PARP-1, the most well characterized PARP, is activated by DNA damage and plays a role in DNA repair. Since increased activity of PARP depletes the intracellular pool of NAD^+^, this may lead to reduced SIRT1 activity and cell death.^57^ Aside from potential effects on SIRT1, the activity of PARP-1 was shown to be rhythmic over the circadian day/night cycle,^58^ resulting in a number of molecular consequences. PARP-1 was shown to directly bind CLOCK and BMAL1 and, subsequently, poly(ADP-ribosyl)ate CLOCK, which also modulated the ability of the circadian transcription factors to bind target DNA consensus sites.^58^ Furthermore, data also suggested that PARP-1 is a critical regulator of feeding entrainment on peripheral circadian clocks. PARP-1–deficient mice, compared to wild-type control animals, exhibited a phase delay in circadian gene expression in response to altered feeding regimens, implying that a link exists between PARP-1 and metabolic cues that signal to peripheral circadian clocks.^58^ Increasing evidence indicates that circadian regulatory enzymes link metabolism with clock-timing systems, yet the extent to which these circuits are regulated by the cellular metabolic state and how they potentially feedback to the central clock is an intriguing concept that requires further investigation.

Given the direct control of SIRT1 deacetylase activity, as well as the control of PARP-1 activity by NAD^+^, circadian regulation of NAD^+^ levels appears to be a critical regulatory mechanism controlling circadian rhythms, metabolism, and cell growth. Interestingly, altered NAMPT levels have been implicated in metabolic disorders and cancer, and FK866, a highly specific NAMPT inhibitor that abolishes NAD^+^ circadian oscillations and thereby SIRT1 cyclic activity, is used to control cell death in human cancer tissues. These results suggest that a direct molecular coupling exists between the circadian clock, energy metabolism, and cell survival. Future studies will reveal the precise function of SIRT1-directed circadian control in regulation of metabolism.

The evidence that NAD^+^ intracellular levels are under control of the circadian clock begs the question regarding what other metabolites also follow a circadian oscillation. Recent studies have reported that in human^59^ and mouse blood plasma,^60^ as well as in mouse liver,^61^ a number of metabolites are under the control of the circadian clock. In mouse liver, varying metabolites peak at different times during the circadian cycle, including nucleotide, carbohydrate, and lipid metabolite peaks at zeitgeber time (ZT) 9, versus amino acid and xenobiotic metabolite peaks at ZT 15–21.^61^ Amino acids and metabolites of the urea cycle were reported to follow a circadian oscillation in mouse plasma.^60^ Also, approximately ∼15% of identified metabolites in human blood plasma or saliva followed a rhythmic pattern, including fatty acids in plasma and amino acids in saliva.^59^ These studies reveal the vast amount of data that are currently unexplored related to circadian metabolomics, and suggest a number of mechanistic pathways remain to the elucidated that connect the circadian clock with metabolic state. Also, considering a number of enzymes (including histone modifiers) that use varying cofactors and metabolites, a detailed analysis of such metabolites that are currently not known to oscillate is needed.

## Concluding remarks

The circadian clock comprises a hierarchical network of transcriptional, translational, and post-translational events that govern a tightly controlled timekeeping system. The intricate interrelationship between the central and peripheral clocks is a highly regulated system that requires precise specificity to maintain proper biological rhythms. Yet emerging evidence suggests that the circadian clock machinery is extremely plastic and can respond to external cues, suggesting an intimate link between the circadian cycle and environmental state. An additional level of complexity exists in the number of metabolic processes that are emerging as direct targets of the circadian machinery. New evidence also suggests that the metabolic state of the cell can directly modulate the circadian epigenome and transcriptional control. We are only beginning to understand the complexity of the circadian clock and appreciate the scope of this network, how it is regulated, and the extent to which it governs systemic physiological state.
